# Responsible attention: the effect of divided attention on metacognition and responsible remembering

**DOI:** 10.1007/s00426-022-01711-w

**Published:** 2022-07-15

**Authors:** Dillon H. Murphy, Alan D. Castel

**Affiliations:** Department of Psychology, University of California, Los Angeles, CA 90095 USA

## Abstract

We are frequently exposed to situations where we need to remember important information when our attentional resources are divided; however, it was previously unclear how divided attention impacts responsible remembering: selective memory for important information to avoid consequences for forgetting. In the present study, we examined participants’ memory for valuable information, metacognitive accuracy, and goal-directed cognitive control mechanisms when under full and divided attention. In Experiment 1, participants were presented with words paired with point values counting towards their score if recalled but were required to “bet” on whether they would remember it. Results revealed that selective memory for high-value information was impaired under divided attention. In Experiment 2, we presented participants with unassociated word pairs and solicited metacognitive predictions of recall (i.e., JOLs). Results revealed that the relative accuracy of participants’ metacognitive judgments was enhanced when studying under divided attention. Experiment 3 examined cognitive control mechanisms to selectively remember goal-relevant information at the expense of information that could potentially be offloaded (i.e., *responsible forgetting*). Results revealed that participants’ ability to strategically prioritize goal-relevant information at the expense of information that could be offloaded was preserved under divided attention. Collectively, *responsible attention* encompasses how attentional resources impact one’s ability to engage in responsible remembering and we demonstrate that responsible remembering can be impaired, enhanced, and preserved in certain contexts.

## Introduction

There are many situations in which we attempt to focus our attention on two or more tasks at once. Listening to music while studying or working on homework, eating while in a class or a meeting, and texting while driving all illustrate instances where we divide our attentional resources. Although some people believe that they are good “multitaskers,” the effects of divided attention on encoding and later memory are detrimental to learning (Castel & Craik, 2003; Craik et al., 1996; Naveh-Benjamin et al., 2000). Despite the well-known negative effects of divided attention on many types of memory (e.g., Baddeley et al., 1984; Castel & Craik, 2003; Craik et al., 1996, 2018; Carrier et al., 2015; Greene & Naveh-Benjamin, 2021; Naveh-Benjamin et al., 1998, 2003; see also Calderwood et al., 2014), learners often multitask, even when studying important information.

Although divided attention generally impairs memory, previous work has demonstrated that the selective prioritization of valuable information (i.e., selectivity; see Castel et al., 2002) can be preserved in some situations when attention is divided (Middlebrooks et al., 2017; Siegel & Castel, 2018; but see Elliott & Brewer, 2019; Siegel et al., 2021) as well as in other conditions that are detrimental to memory performance like insufficient study time (Middlebrooks et al., 2016). This prioritization of valuable information, especially in conditions where memory is impaired, exemplifies *responsible remembering*: one’s knowledge about selective memory processes allowing for the efficient use of memory to remember important information in a variety of contexts (Murphy & Castel, 2020, 2021a, b, 2022a; Murphy et al., 2022a).

Responsible remembering captures how our memory functions to prioritize important information that will need to be remembered as well as how metacognitive processes may be more precise in situations involving consequences for forgetting. For example, Murphy and Castel (2021a; see also Murphy et al., 2022a) asked participants to remember sets of children and their associated food preferences (including foods the kids were allergic to). When participants were forced to consider the importance of remembering each child’s food preferences, information with consequences for forgetting was deemed most important and subsequently best remembered. Thus, if people learn to self-assess and prioritize information that will need to be remembered or have negative consequences if forgotten, the recall of said important information can be enhanced. However, situations involving responsible remembering often involve environments full of distractions (i.e., watching TV while babysitting) and people need to learn and remember important information while distracted. Additionally, although it may be responsible to avoid distractions and allocate all of one’s cognitive resources towards remembering the most important information, distractions are often unavoidable and some learners frequently divide their attention despite the well-known effects of divided attention on memory (Fried, 2008; Sana et al., 2013).

In addition to harming memory, divided attention may also negatively affect metacognition (i.e., the awareness of our memory processes; Nelson & Narens, 1990; see also Dunlosky et al., 2016; Nelson, 1996; Rhodes, 2016). To examine participants’ metacognitive awareness of their learning (i.e., metacognitive monitoring), researchers often solicit judgments of learning (JOLs) whereby learners predict the likelihood of remembering studied information. Although JOLs are often accurate and learners are generally aware of their selective memory for valuable information (e.g., Murphy et al., 2021; see also Murphy et al., 2022b), previous work on the effect of divided attention on metacognitive judgments indicates that divided attention may hinder some metacognitive mechanisms (e.g., Barnes & Dougherty, 2007; Konishi et al., 2021; Sacher et al., 2009, 2013). Specifically, learners’ global predictions of performance generally account for decreased memory under divided attention, but participants are sometimes overconfident when making item-level judgments (Beaman et al., 2014; Kelley & Sahakyan, 2003; Sacher et al., 2009). However, recent work suggests that metacognitive monitoring can be largely preserved under divided attention (e.g., Peng & Tullis, 2021; see Peng & Tullis, 2022 for a review). Thus, with fewer attentional resources available, there may be situations where participants’ ability to monitor their learning is preserved and others where it is impaired, potentially impacting the ability to engage in responsible remembering.

The possible detrimental effects of divided attention on memory could also extend to learners’ ability to execute a value-based agenda for later remembering (see agenda-based regulation; Ariel, 2013; Ariel & Dunlosky, 2013; Ariel et al., 2009; Dunlosky & Ariel, 2011a, b; Dunlosky et al., 2011). Specifically, the ability to develop and use goal-oriented agendas to strategically focus on important information, an attentionally demanding process, may be impaired if participants have fewer attentional resources available. Furthermore, the method by which selectivity for valuable information is often achieved, deep semantic processing (Cohen et al., 2014; Hennessee et al., 2019), tends to be more susceptible to the effects of divided attention (Anderson et al., 2000; Craik, 1982). Thus, responsible remembering may require a full allotment of attentional resources or in conditions where attention is divided, the functional prioritization of attention to counteract the costs of reduced attentional resources, a facet of responsible remembering we are calling *responsible attention*.

Since the most important or valuable information is often associated with the most severe outcomes if forgotten, situations involving consequences for forgetting often lead to improved metacognition and learning outcomes (e.g., McGillivray & Castel, 2011). Additionally, when attentional resources are spent on a competing task, learners’ remaining resources should be devoted to engaging in responsible remembering. Specifically, when attention is divided, metacognitive mechanisms may engage the learner’s awareness of the need to selectively remember and participants can compensate for the limitations of divided attention by devoting their remaining cognitive resources to the most critical information (Middlebrooks et al., 2017). In the current study, we examined the encoding and later remembering of important information or information with consequences if forgotten, the accuracy of metacognition, and cognitive control mechanisms (i.e., the ability to engage in functional, goal-directed behavior; see Chiew & Braver, 2017; Diamond, 2013; Egner, 2017; Miller & Cohen, 2001) that contribute to responsible remembering when fewer attentional resources are available during encoding. In each experiment, we assessed whether participants were less able to engage in responsible remembering when under divided attention, potentially indicating that a full allotment of attentional resources during encoding is crucial for remembering important information.

## The current study

To determine the role of attention in responsible remembering, we examined participants’ memory for information with consequences for forgetting, metacognitive accuracy, and the strategic remembering and forgetting of information according to one’s goals when under full and divided attention. In Experiment 1, to simulate a situation with consequences for misguided metacognition, participants completed a value-directed remembering task where they were required to “bet” on whether they would later remember each word. We expected participants under divided attention to demonstrate more strategic betting behavior by strategically betting on and better remembering valuable words, leading to preserved selectivity (similar to older adults, see McGillivray & Castel, 2011). In Experiment 2, we presented participants with word pairs while either under full or divided attention to elucidate how divided attention impacts the metacognitive accuracy of JOLs. Finally, in Experiment 3, we investigated the role of strategic forgetting (i.e., a form of *responsible forgetting*, see Murphy & Castel, 2021b, 2022a) when under divided attention to determine if participants are less likely to remember goal-relevant information at the expense of potentially offloaded information when under divided attention.

## Experiment 1

In Experiment 1, participants completed a value-directed remembering task where they were presented with lists of words paired with point values that count towards their score if recalled (see Castel et al., 2002). After the presentation of each word, participants predicted if they would later recall each word by “betting” the points associated with each word (adapted from McGillivray & Castel, 2011). If participants bet on and successfully recalled a word, they received the associated points but if they failed to recall the word, they lost the associated points. Thus, this paradigm examined the impact of item importance, feedback, and experience on metacognitive judgments and accuracy. Participants either completed the study phase under full or divided attention and we expected participants with fewer attentional resources to demonstrate preserved selectivity (Middlebrooks et al., 2017), as this has also been found for older adults under full attention (McGillivray & Castel, 2011). Thus, we wanted to determine if younger adults under divided attention could strategically prioritize valuable information and engage in responsible remembering after gaining task experience (i.e., after experiencing instances of forgetting valuable information on early lists).

### Method

#### Participants

Participants were 96 undergraduate students (*M*_age_ = 19.32, SD_age_ = 1.73) recruited from the University of California Los Angeles (UCLA) Human Subjects Pool. Participants were tested online and received course credit for their participation. Participants were excluded from analysis if they admitted to cheating (e.g., writing down answers) in a post-task questionnaire (they were told they would still receive credit if they cheated). This exclusion process resulted in zero exclusions. Participants were also excluded for failing to complete the divided attention task with at least 50% accuracy (similar to prior work; see Siegel & Castel, 2018; Siegel et al., 2021). This exclusion process resulted in nine exclusions. A power analysis was conducted using G*Power (Faul et al., 2007). For a 2 (attention: full, divided) × 8 (list) mixed ANOVA, assuming alpha = 0.05, power = 0.80, and that outcomes on each list are highly correlated (*r* = 0.50; based on prior work using a similar design, see Murphy et al., 2021), 72 participants would be needed to reliably detect small differences (since prior work did not find significant differences, see Middlebrooks et al., 2017) between participants under full and divided attention (*η*_*p*_^2^ = 0.06).

#### Materials and procedure

Participants completed a value-directed remembering task (where words were paired with point values) with a metamemory “betting” component (adapted from McGillivray & Castel, 2011). For each word, participants had to decide if they wanted to “bet” on it. If the participant said “yes” (they did want to bet on it) and they later recalled that word, they received the associated points. However, if participants failed to recall a word that they initially bet on, then they lost those points. Conversely, if the participant said “no” (they did not want to bet on it), points were not gained or lost regardless of whether the word was later recalled. Whether the participant chose to bet on the word or not, each word was displayed for 5 s. Point values (1–10, 15, and 20) were randomly paired with words within each list. The inclusion of the 15 and 20 point values was to assess the impact of extreme incentive or loss potential (e.g., Loftus & Wickens, 1970). Participants were told that the goal was to try to get as many points as possible and were encouraged to try to maximize gains and minimize any losses.

The studied words were between 4 and 6 letters (*M* = 4.61, SD = 0.55). In terms of concreteness (with lower values indicating lower concreteness and higher values indicating higher concreteness), words ranged from 3.07 to 5.00 and averaged a score of 4.66 (SD = 0.33). On the log-transformed Hyperspace Analogue to Language (HAL) frequency scale (with lower values indicating lower frequency in the English language and higher values indicating higher frequency), words ranged from 5.27 to 12.47 and averaged a score of 8.71 (SD = 1.23). Words were classified according to the English Lexicon Project website (Balota et al., 2007).

Following the presentation of the 12 words on each list, participants completed an immediate 30-s free recall test. Scores were calculated by summing the points associated with the words participants bet on and recalled and then subtracting the number of points associated with the words that were bet on but not recalled. Immediately following the recall period, participants were informed of their score for that list but were not given feedback about specific items. This procedure was repeated for eight study-test cycles.

Participants either completed the study phase under full (*n* = 51) or divided attention (*n* = 45). Participants in the divided-attention condition were told that they would hear a series of low-pitched (400 Hz) and high-pitched (900 Hz) tones during the study phase. A tone was played every 3 s with each tone lasting 1 s. Tone sequences were randomly generated for each participant. Participants were instructed to indicate (on the keyboard) whether each pitch they heard was low or high and the text “awaiting tone response” would appear on the screen if participants did not respond to the tones. Participants completed a short tone discrimination practice session before beginning the task.

### Results

On the divided attention task, participants correctly identified an average of 77.7% of the tones (SD = 0.13) on each list. To examine performance on the secondary task as a function of list, a within-subjects ANOVA with eight levels (list) was conducted but Mauchly’s test of sphericity indicated violations for list [Mauchly’s *W* = 0.26, *p* < 0.001]. Huynh–Feldt corrected results revealed a main effect of list [*F*(5.78, 254.38) = 6.50, *p* < 0.001, *η*_*p*_^2^ = 0.13] such that performance on the secondary task increased after the first list. Pearson correlations between divided attention task performance, the proportion of words bet on, the proportion of words recalled, and point scores are shown in Table 1.

**Table 1:** Pearson (*r*) correlations between divided attention task performance, the proportion of words bet on, the proportion of words recalled, and point scores in Experiment 1

Measure	1	2	3	4
1. Divided Attention Performance	–			
2. Proportion of Words Bet On	− 0.010	–		
3. Proportion of Words Recalled	0.146	0.690***	–	
4. Point Scores	0.215	0.165	− 0.624***	–

*p < 0.05, **p < 0.01, ***p < 0.001

To investigate differences in recall performance as a function of attention during encoding, a 2 (attention: full, divided) × 8 (list) mixed ANOVA did not reveal a main effect of list [Mauchly’s *W* = 0.45, *p* < 0.001: Huynh–Feldt corrected results: *F*(6.09, 572.60) = 0.82, *p* = 0.560, *η*_*p*_^2^ = 0.01] such that the proportion of words recalled on each list did not change with increased task experience. However, there was a main effect of attention [*F*(1, 94) = 6.85, *p* = 0.010, *η*_*p*_^2^ = 0.07] such that participants with full attention (*M* = 0.51, SD = 0.17) recalled more words than participants under divided attention (*M* = 0.44, SD = 0.11). Additionally, list interacted with attention [*F*(6.09, 572.60) = 3.36, *p* = 0.003, *η*_*p*_^2^ = 0.03] such that recall for participants under divided attention improved with increased task experience.

To examine differences in scores (sum of the values of recalled words that were bet on minus the values of words that were bet on and not recalled) as a function of attention during encoding, a 2 (attention: full, divided) × 8 (list) mixed ANOVA revealed a main effect of list [*F*(7, 658) = 6.62, *p* < 0.001, *η*_*p*_^2^ = 0.07] such that scores increased with task experience. Additionally, there was a main effect of attention [*F*(1, 94) = 5.96, *p* = 0.016, *η*_*p*_^2^ = 0.06] such that participants with full attention (*M* = 45.28, SD = 21.56) had greater scores than participants under divided attention (*M* = 35.16, SD = 18.69). However, list did not interact with attention [*F*(7, 658) = 1.58, *p* = 0.138, *η*_*p*_^2^ = 0.02].

To investigate differences in the proportion of words bet on as a function of attention during encoding, a 2 (attention: full, divided) × 8 (list) mixed ANOVA did not reveal a main effect of list [Mauchly’s *W* = 0.27, *p* < 0.001; Huynh–Feldt corrected results: *F*(5.41, 508.69) = 1.34, *p* = 0.242, *η*_*p*_^2^ = 0.01]. However, there was a main effect of attention [*F*(1, 94) = 5.75, *p* = 0.018, *η*_*p*_^2^ = 0.06] such that participants with full attention (*M* = 0.55, *SD* = 0.18) bet on a greater proportion of words than participants under divided attention (*M* = 0.47, SD = 0.15). Moreover, list did not interact with attention [*F*(5.41, 508.69) = 0.65, *p* = 0.677, *η*_*p*_^2^ = 0.01].

To determine if participants attempted to be selective in their betting behavior, we computed a multilevel model (MLM) where we treated the data as hierarchical or clustered (i.e., multilevel) with items nested within individual participants. Since betting at the item level was binary (bet or no bet), we conducted logistic MLMs to examine betting behavior. In these analyses, the regression coefficients are given as logit units (i.e., the log odds of betting). We report exponential betas (*e*^B^), and their 95% confidence intervals, which give the coefficient as an odds ratio (i.e., the odds of betting divided by the odds of not betting). Thus, *e*^B^ can be interpreted as the extent to which the odds of betting changed. Specifically, values greater than 1 represent an increased likelihood of betting while values less than 1 represent a decreased likelihood of betting.

**Fig. 1:**
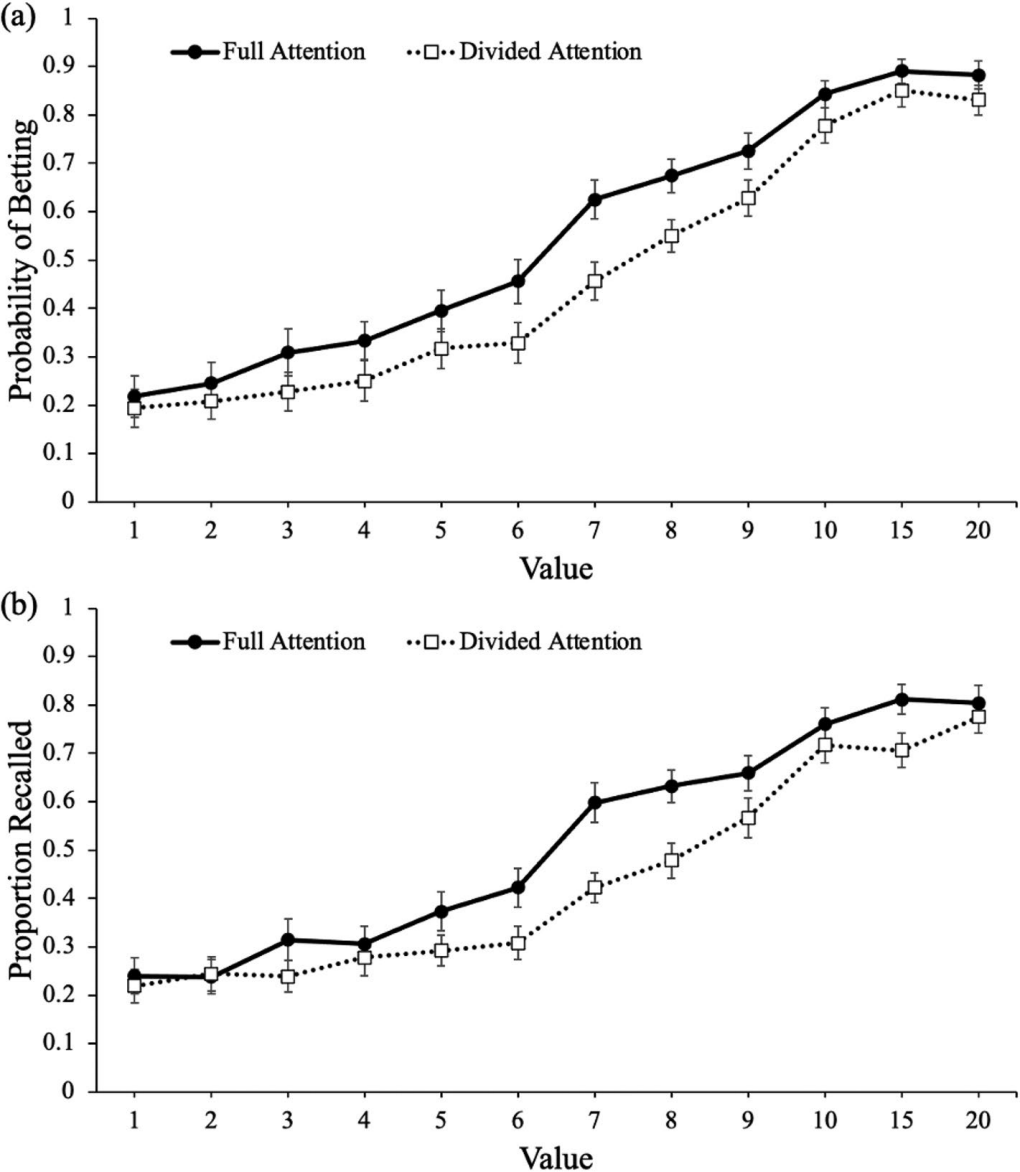
Probability of betting on a word (**a**) and recalling a word (**b**) as a function of value and attention in Experiment 1. Error bars reflect the standard error of the mean

To examine betting behavior, a logistic MLM with item-level betting modeled as a function of value with attention at encoding (full, divided) as a between-subjects factor revealed that value significantly predicted betting decisions [*e*^B^ = 1.30, CI: 1.28–1.32, *z* = 38.27, *p* < 0.001] such that high-value words were bet on more than low-value words. Additionally, attention significantly predicted betting decisions [*e*^B^ = 1.74, CI: 1.15–2.61, *z* = 2.64, *p* = 0.008] and value interacted with attention [*e*^B^ = 1.04, CI: 1.01–1.07, *z* = 2.71, *p* = 0.007] such that value was a stronger predictor of betting decisions for participants with full attention compared with participants under divided attention (see Fig. 1a). Specifically, post hoc MLMs with value predicting betting revealed that value was a better predictor of betting for participants with full attention [*e*^B^ = 1.33, CI: 1.30–1.35, *z* = 27.80, *p* < 0.001] than for participants under divided attention [*e*^B^ = 1.27, CI: 1.25–1.30, *z* = 26.29, *p* < 0.001].

Finally, to determine if participants were selective (see Fig. 1b), we conducted a logistic MLM with item-level recall accuracy modeled as a function of value with attention at encoding (full, divided) as a between-subjects factor. Since this model examined recall accuracy, *e*^B^ can be interpreted as the extent to which the odds of correctly recalling a word changed. Specifically, values greater than 1 represent an increased likelihood of being correct while values less than 1 represent a decreased likelihood of being correct. Results revealed that value significantly predicted recall [*e*^B^ = 1.20, CI: 1.19–1.22, *z* = 33.94, *p* < 0.001] such that high-value words were recalled better than low-valued words. Additionally, attention significantly predicted recall [*e*^B^ = 1.52, CI: 1.12–2.06, *z* = 2.67, *p* = 0.008] and value interacted with attention [*e*^B^ = 1.03, CI: 1.01–1.05, *z* = 2.92, *p* = 0.003] such that value was a better predictor of recall for participants with full attention than participants under divided attention, indicating impaired selectivity. Specifically, post-hoc MLMs with value predicting recall revealed that value was a better predictor of recall for participants with full attention [*e*^B^ = 1.22, CI: 1.20–1.24, *z* = 25.23, *p* < 0.001] than for participants under divided attention [*e*^B^ = 1.18, CI: 1.17–1.20, *z* = 22.68, *p* < 0.001].

### Discussion

In Experiment 1, participants under divided attention made fewer bets, recalled fewer words, and had lower scores than participants under full attention. Additionally, both the likelihood of betting on and recalling high-value words was impaired for participants under divided attention relative to participants with full attention. As previously mentioned, the task used in Experiment 1 was adapted from prior work examining the role of rewards and punishments for forgetting in younger and older adults (McGillivray & Castel, 2011). In this previous work, despite recalling fewer words overall, older adults were able to compensate for their recall deficit by making more responsible and strategic betting decisions as well as selectively best remembering the highest valued words as they gained task experience. However, in the present study, dividing younger participants’ attention hindered their ability to engage in this form of responsible remembering indicating that full attentional resources may be an essential component of some forms of responsible remembering.

Although some prior work indicates that certain forms of selectivity may be preserved under divided attention (Middlebrooks et al., 2017), more recent work suggests that selectivity is impaired when attention is divided at encoding (e.g., Elliott & Brewer, 2019; Siegel et al., 2021). Experiment 1 provides further evidence that when learners’ attention is divided, their ability to engage in goal-directed behaviors to maximize memory utility is impaired. Specifically, attentional resources may be a crucial component of strategic encoding processes (but retrieval processes also play a role, see Murphy & Castel, 2022b; Stefanidi et al., 2018) and in the presence of competing task demands, participants with fewer attentional resources available may employ less strategic encoding mechanisms (see Murphy et al., 2022c for an example of strategic processing) compared with participants with a full allotment of attentional resources, resulting in poorer memory outcomes.

## Experiment 2

In Experiment 1, dividing participants’ attention when there were consequences for forgetting impaired their ability to engage in responsible remembering. In Experiment 2, rather than examining how selectivity for valuable information is affected under divided attention, we investigated how participants’ ability to monitor their learning is impacted when fewer attentional resources are available. Specifically, rather than making binary betting predictions of remembering, we presented participants with unassociated word pairs and solicited JOLs for each pair. Previous work has indicated that learners have some awareness that memory suffers under divided attention (Barnes & Dougherty, 2007; Finley et al., 2014; Junco & Cotten, 2011; Peng & Tullis, 2021, 2022) but we expected participants under divided attention to demonstrate poorer metacognitive accuracy under divided attention (see Beaman et al., 2014; Kelley & Sahakyan, 2003; Sacher et al., 2009).

### Method

#### Participants

After exclusions, participants were 85 undergraduate students (*M*_age_ = 20.40, SD_age_ = 1.66) recruited from the UCLA Human Subjects Pool. Participants were tested online and received course credit for their participation. Participants were excluded from analysis if they admitted to cheating (e.g., writing down answers) in a post-task questionnaire (they were told they would still receive credit if they cheated). This exclusion process resulted in four exclusions. Participants were also excluded for failing to complete the divided attention task with at least 50% accuracy. This exclusion process resulted in 19 exclusions. A power analysis indicated that for a 2 (attention: full, divided) × 6 (list) mixed ANOVA, assuming alpha = 0.05, power = 0.80, and that outcomes on each list are highly correlated (*r* = 0.60; based on pilot data using a similar design), 84 participants would be needed to reliably detect small differences (since some prior work did not find significant differences, see Peng & Tullis, 2021) between participants under full and divided attention (*η*_*p*_^2^ = 0.06).

#### Materials and procedure

Participants were told that they would be presented with several word pairs to remember (e.g., table-fan) and after each list of word pairs was presented, they would be tested on those word pairs. Participants were presented with 6 lists of 20 word pairs and each pair was shown one at a time, for 5 s each, in random order. The stimulus words were between 4 and 7 letters (*M* = 5.05, SD = 0.98). In terms of concreteness, words ranged from 2.50 to 5.00 and averaged a score of 4.52 (SD = 0.47). On the log-transformed Hyperspace Analogue to Language (HAL) frequency scale, words ranged from 5.48 to 10.96 and averaged a score of 8.56 (SD = 1.40). After the presentation of each pair, participants made a judgment of how likely they were to later remember it (JOL) on a scale from 0 (meaning they definitely would not remember the pair) to 100 (meaning they definitely would remember the pair). After each list was presented, participants were shown the left word from each pair one at a time, in random order, and asked to recall the associated word. Participants were given as much time as they needed to recall each pair. Participants either completed the study phase under full (*n* = 45) or divided attention (*n* = 40). The divided attention task was similar to Experiment 1, but the tones did not play while participants made their JOL for each word. Thus, attention was only divided during encoding and not while participants provided their JOLs.

#### Results

On the divided attention task, participants correctly identified an average of 87.2% of the tones (SD = 0.12) on each list. To examine performance on the secondary task as a function of list, a within-subjects ANOVA with 6 levels (list) was conducted. However, results did not reveal a main effect of list [Mauchly’s *W* = 0.05, *p* < 0.001; Huynh–Feldt corrected results: *F*(3.17, 123.64) = 1.43, *p* = 0.235, *η*_*p*_^2^ = 0.04]. Pearson correlations between divided attention task performance, average judgments, the proportion of words recalled, calibration, and resolution are shown in Table 2.

**Table 2:** Pearson (*r*) correlations between divided attention task performance, average judgments, the proportion of words recalled, calibration, and resolution in Experiment 2

Measure	1	2	3	4	5
1. Divided Attention Performance	–				
2. Average JOL	0.236	–			
3. Proportion of Words Recalled	0.204	0.659***	–		
4. Resolution	− 0.043	− 0.226*	− 0.324**	–	
5. Calibration	0.029	0.264*	− 0.551***	0.172	–

*p < 0.05, **p < 0.01, ***p < 0.001

To examine differences in judgments as a function of attention during encoding, a 2 (attention: full, divided) × 6 (list) mixed ANOVA revealed a main effect of list [Mauchly’s *W* = 0.050, *p* < 0.001; Huynh–Feldt corrected results: *F*(2.23, 185.34) = 9.87, *p* < 0.001, *η*_*p*_^2^ = 0.11] such that JOLs decreased as the task endured. Additionally, there was a main effect of attention [*F*(1, 83) = 4.02, *p* = 0.048, *η*_*p*_^2^ = 0.05] such that, on average, participants with full attention (*M* = 42.76, SD = 19.74) expected that they were more likely to recall each word pair than participants under divided attention (*M* = 33.56, SD = 21.58). Moreover, there was a significant interaction between list and attention [*F*(2.23, 185.34) = 3.43, *p* = 0.030, *η*_*p*_^2^ = 0.04] such that judgments showed a greater decrease on later lists in participants under divided attention.

To investigate differences in recall performance as a function of attention during encoding, a 2 (attention: full, divided) × 6 (list) mixed ANOVA revealed a main effect of list [Mauchly’s *W* = 0.13, *p* < 0.001; Huynh–Feldt corrected results: *F*(2.66, 220.51) = 5.31, *p* = 0.002, *η*_*p*_^2^ = 0.06] such that the proportion of word pairs recalled on each list increased as the task endured. There was also a main effect of attention [*F*(1, 83) = 4.59, *p* = 0.035, *η*_*p*_^2^ = 0.05] such that participants with full attention (*M* = 0.55, SD = 0.25) recalled more word pairs than participants under divided attention (*M* = 0.44, SD = 0.22). Additionally, list interacted with attention [*F*(2.66, 220.51) = 5.47, *p* = 0.002, *η*_*p*_^2^ = 0.06] such that participants with full attention recalled more word pairs per list with increased task experience.

Since JOLs were assessed as a percentage likelihood (same scale as the probability of recall), we computed measures of *absolute* and *relative accuracy* (see Higham et al., 2016; Rhodes, 2016). Absolute accuracy (i.e., *calibration*) is the overall relationship between judgments and performance and is computed as the difference between a participant’s average judgments and the percentage of items recalled. Positive scores indicate overconfidence while negative scores indicate underconfidence; scores of zero indicate perfect calibration (a direct correspondence between predictions and recall).

**Fig. 2:**
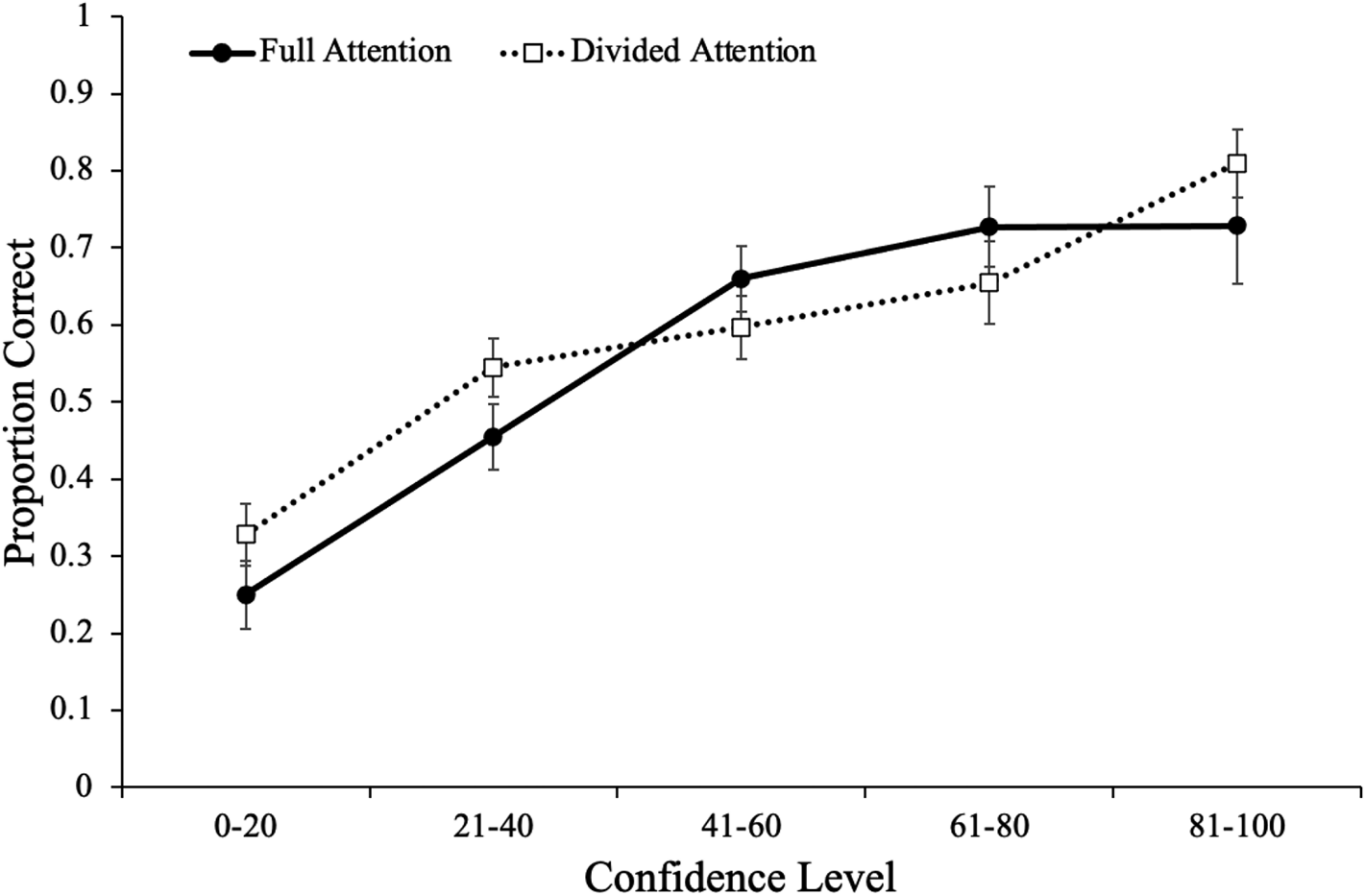
Judgment-specific accuracy assessed with judgment-accuracy characteristic curves as a function of attention at encoding in Experiment 2. Error bars reflect the standard error of the mean

Figure 2 displays calibration curves (broken into five bins: 0–20, 21–40, 41–60, 61–80, 81–100) for participants under full and divided attention. To investigate differences in calibration as a function of attention during encoding, a 2 (attention: full, divided) × 6 (list) mixed ANOVA revealed a main effect of list [Mauchly’s *W* = 0.24, *p* < 0.001; Huynh–Feldt corrected results: *F*(3.23, 268.18) = 11.95, *p* < 0.001, *η*_*p*_^2^ = 0.13] such that participants became more underconfident with increased task experience. However, there was not a main effect of attention [*F*(1, 83) = 0.25, *p* = 0.616, *η*_*p*_^2^ < 0.01] such that participants with full attention (*M* = − 11.87, SD = 21.30) were similarly calibrated as participants under divided attention (*M* = − 9.79, SD = 16.12). Moreover, list did not interact with attention [*F*(3.23, 268.18) = 2.52, *p* = 0.054, *η*_*p*_^2^ = 0.03].

*Relative accuracy* (i.e., *resolution*) is the extent to which judgments discriminate between information that is or is not remembered and is often measured by Gamma correlations between JOLs and recall accuracy for each item for each participant (see Masson & Rotello, 2009 for alternative approaches). A high correlation between judgments and performance would exemplify the ability to distinguish between what will or will not be remembered; the individual remembers items given high JOLs and forgets items given low JOLs.

**Fig. 3:**
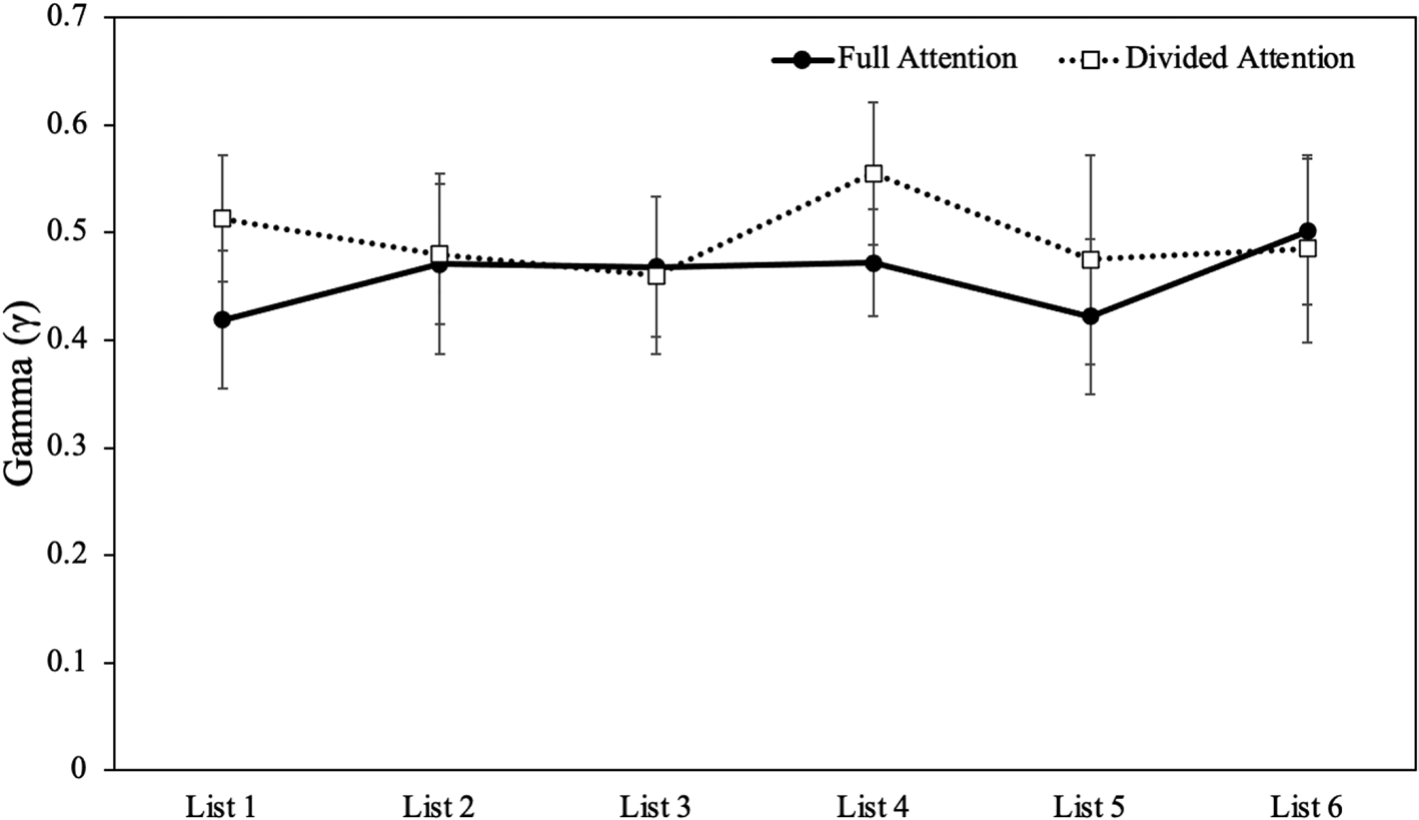
Gamma (*γ*) correlations between judgments and recall as a function of list in Experiment 2. Error bars reflect the standard error of the mean

We computed Gamma correlations for each participant and these correlations served as the dependent variable in a 2 (attention: full, divided) × 6 (list) mixed ANOVA (see Fig. 3). Results did not reveal a main effect of list [Mauchly’s *W* = 0.50, *p* < 0.001; Huynh–Feldt corrected results: *F*(4.26, 230.04) = 0.44, *p* = 0.794, η_p_^2^ = 0.01] such that relative accuracy did not change with task experience. However, there was a main effect of attention [*F*(1, 54^1^) = 6.58, *p* = 0.013, *η*_*p*_^2^ = 0.11] such that participants with full attention (*M* = 0.36, SD = 0.36) were less relatively accurate than participants under divided attention (*M* = 0.51, SD = 0.33). Moreover, list did not interact with attention [*F*(4.26, 230.04) = 0.45, *p* = 0.781, *η*_*p*_^2^ = 0.01]. In addition to examining Gamma correlations at the participant level, we also examined overall Gamma correlations for each group. Again, results revealed that participants under divided attention (*γ* = 0.60, *p* < 0.001) showed better relative accuracy than participants with full attention (*γ* = 0.45, *p* < 0.001).

In addition to analyzing Gamma correlations, we also conducted a logistic MLM (see Murayama et al., 2014 for benefits of this approach) with item-level recall accuracy modeled as a function of JOLs with attention at encoding (full, divided) as a between-subjects factor. Results revealed that judgments significantly predicted recall [*e*^B^ = 1.04, CI: 1.03–1.04, *z* = 29.49, *p* < 0.001] such that words given higher JOLs were recalled better than words given lower JOLs. However, attention did not significantly predict recall [*e*^B^ = 1.33, CI: 0.86–2.06, *z* = 1.28, *p* = 0.201] but JOLs interacted with attention [*e*^B^ = 0.99, CI: 0.98–0.99, *z* = − 5.61, *p* < 0.001] such that JOLs better predicted recall for participants under divided attention, indicating enhanced metacognitive accuracy. Specifically, post hoc MLMs with JOLs predicting recall revealed that JOLs were a better predictor of recall for participants under divided attention [*e*^B^ = 1.04, CI: 1.04–1.05, *z* = 23.37, *p* < 0.001] than for participants with full attention [*e*^B^ = 1.03, CI: 1.03–1.03, *z* = 18.05, *p* < 0.001].

#### Discussion

In Experiment 2, participants under divided attention gave lower JOLs and demonstrated poorer recall than participants under full attention. This resulted in similar calibration between participants under full and divided attention; however, in terms of resolution (as measured by both Gamma correlations and MLMs), participants under divided attention were more relatively accurate than participants with full attention. Thus, in contrast to some prior work on the effect of divided attention on metacognition (e.g., Beaman et al., 2014; Kelley & Sahakyan, 2003; Konishi et al., 2021; Sacher et al., 2009), Experiment 2 demonstrates that when fewer attentional resources are available during encoding, the accuracy of metacognitive monitoring can be maintained or potentially improved in some conditions (see also Peng & Tullis, 2021). However, in the present task participants only performed the divided attention task while studying the words—not when making their monitoring judgments. Future work may benefit from including a divided attention task during the JOL phase.

## Experiment 3

In Experiment 1, we demonstrated that some forms of metacognition and selectivity can be impaired when encoding under divided attention while in Experiment 2, we demonstrated that metacognition can be maintained or possibly enhanced when studying information with fewer attentional resources available. In Experiment 3, we were interested in how other cognitive mechanisms that contribute to responsible remembering are affected by divided attention. Specifically, we examined participants’ ability to exert cognitive control to strategically remember goal-relevant information when fewer attentional resources are available.

We presented participants with a list of items to pack for a camping trip with each item followed by a cue indicating whether the participant (“You”) or their friend (“Friend”) was responsible for remembering the word (adapted from Murphy & Castel, 2021b, 2022a). Similar to item-method directed forgetting tasks (see MacLeod, 1998 for a review), we presented the cue indicating whether the participant or their hypothetical friend was responsible for remembering the item after each word appeared so that participants had to process each item; only after having processed the word could participants engaging in the responsible forgetting of items their friend was responsible for remembering (see Murphy & Castel, 2021b).

Participants either completed the study phase under full or divided attention and were then given a free recall test for all of the words, regardless of the cue, as well as a surprise recognition test. While we expected both groups to best remember and recognize items they were responsible for remembering, we expected participants under divided attention to be less sensitive to the cue indicating who was responsible for remembering each word. Alternatively, participants under divided attention may more heavily utilize the opportunity to offload information and show decreased recall and recognition accuracy for friend items compared to participants under full attention.

### Method

#### Participants

After exclusions, participants were 65 undergraduate students (*M*_age_ = 19.95, SD_age_ = 2.20) recruited from the UCLA Human Subjects Pool. Participants were tested online and received course credit for their participation. Participants were excluded from analysis if they admitted to cheating (e.g., writing down answers) in a post-task questionnaire (they were told they would still receive credit if they cheated). This exclusion process resulted in zero exclusions. Participants were also excluded for failing to complete the divided attention task with at least 50% accuracy. This exclusion process resulted in five exclusions. A power analysis indicated that for a 2 (cue: Friend, You) × 2 (attention: full, divided) mixed ANOVA, assuming alpha = 0.05, power = 0.80, and that recall for each cue is not correlated (*r* = 0.01; based on prior work using a similar design, see Murphy & Castel, 2021b), 65 participants would be needed to reliably detect small differences between participants under full and divided attention (*η*_*p*_^2^ = 0.06).

#### Materials and procedure

Participants were told to imagine that they and a (hypothetical) friend were going camping and that they would be presented with a list of items that they and their friend needed to remember to bring on the trip. After each word was presented, a cue indicated whether the participant (“You”) or their friend (“Friend”) was responsible for remembering the word. For each participant, half of the words were randomly designated as to-be-remembered words for the participants, and half were designated as words their friend was responsible for remembering. Each word was preceded by a 1-s fixation cross, then appeared on the screen, one at a time, in random order, for 3 s followed by the cue for an additional 2 s. After the presentation of all 20 words, participants were given a 1-min free recall test in which they were asked to recall all of the words that both they and their friend needed to remember from the just-presented list.

Following the recall test, participants completed a surprise recognition test whereby they were shown the items from the just-presented list as well as 20 lures (in random order) and asked to indicate whether each item was on the list of presented items to bring for the camping trip. Participants indicated their confidence in each response on a scale from 0 to 100 (with 0 being not at all confident and 100 being very confident) and were given as much time as they needed for this portion of the task. Participants either completed the encoding phase under full (*n* = 35) or divided attention (*n* = 30), and the divided attention task was similar to Experiments 1 and 2.

The to-be-remembered words were between 2 and 12 letters (*M* = 5.95, SD = 2.32). In terms of concreteness, words ranged from 4.42 to 5.00 and averaged a score of 4.88 (SD = 0.18). On the log-transformed Hyperspace Analogue to Language (HAL) frequency scale, words ranged from 5.36 to 12.37 and averaged a score of 8.60 (SD = 1.73). The lures were between 3 and 9 letters (*M* = 5.67, SD *=* 1.41). In terms of concreteness, words ranged from 4.14 to 5.00 and averaged a score of 4.74 (SD = 0.24). On the log-transformed Hyperspace Analogue to Language (HAL) frequency scale, words ranged from 6.61 to 11.18 and averaged a score of 8.80 (SD = 1.23).

#### Results

On the divided attention task, participants correctly identified an average of 89.7% of the tones (SD = 0.09). Pearson correlations between divided attention task performance, the proportion of words recalled, recognition, and confidence for You and Friend items are shown in Table 3.

**Table 3:** Pearson (*r*) correlations between divided attention task performance, the proportion of words recalled, recognition, and confidence for You and Friend items in Experiment 3

Measure	1	2	3	4	5	6	7
1. Divided Attention Performance	–						
2. Friend Proportion Recalled	0.105	–					
3. You Proportion Recalled	0.050	0.171	–				
4. Friend A′	− 0.156	0.432***	0.493***	–			
5. You A′	− 0.147	0.293*	0.674***	0.572***	–		
6. Friend Confidence	− 0.226	0.406***	0.349**	0.529***	0.520***	–	
7. You Confidence	− 0.060	0.186	0.586***	0.330**	0.646***	0.714***	–

*p < 0.05, **p < 0.01, ***p < 0.001

**Fig. 4:**
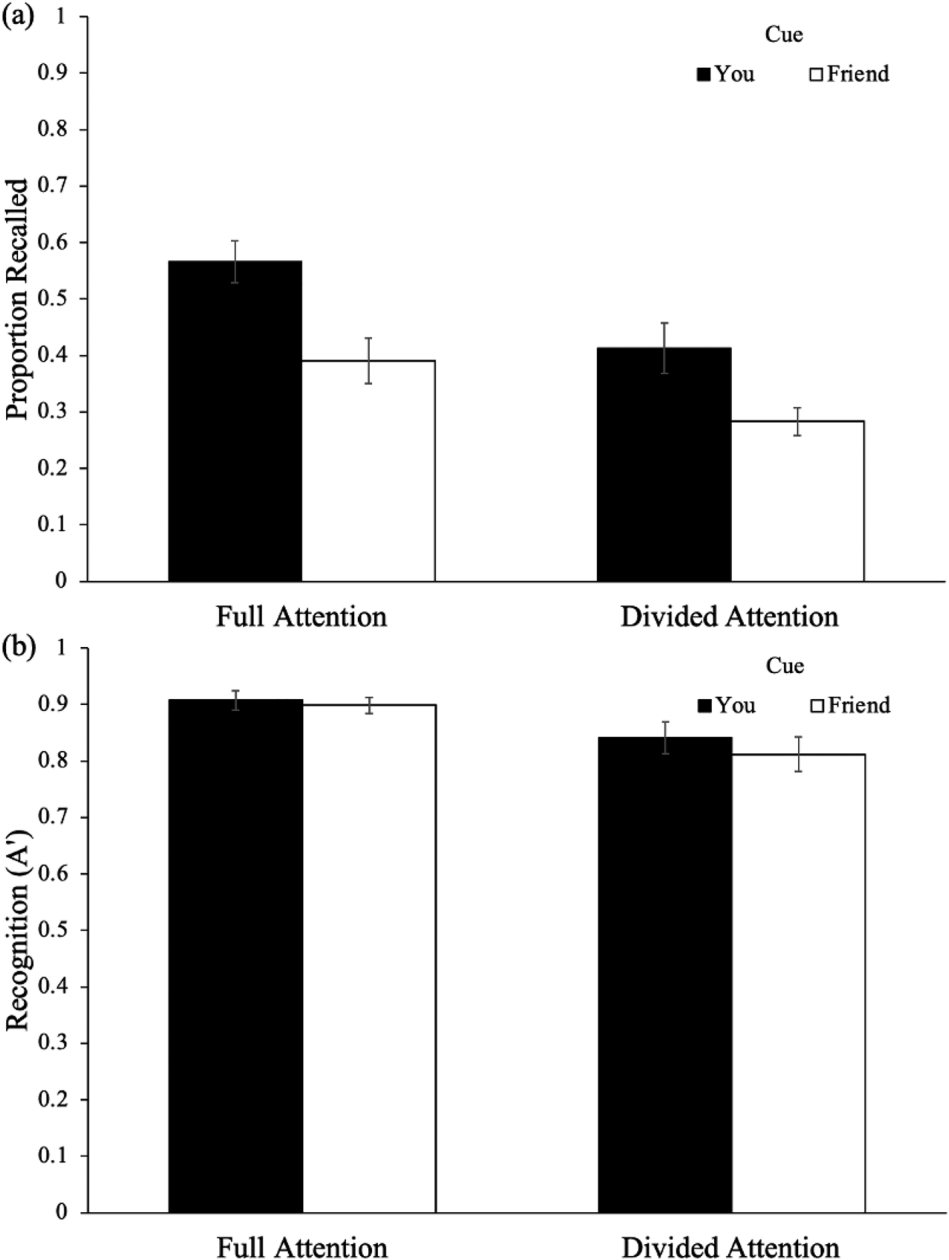
Recall (**a**) and recognition (**b**) performance as a function of cue and attention in Experiment 3. Error bars reflect the standard error of the mean

Recall performance as a function of cue and attention is shown in Fig. 4a. To examine differences in the proportion of words recalled, a 2 (cue: Friend, You) × 2 (attention: full, divided) mixed ANOVA revealed a main effect of cue [*F*(1, 63) = 17.95, *p* < 0.001, *η*_*p*_^2^ = 0.22] such that participants recalled more You items (*M* = 0.50, SD = 0.24) than Friend items (*M* = 0.34, SD = 0.20). Additionally, results revealed a main effect of attention [*F*(1, 63) = 10.93, *p* = 0.002, *η*_*p*_^2^ = 0.15] such that participants with full attention (*M* = 0.48, SD = 0.16) recalled more items than participants under divided attention (*M* = 0.35, SD = 0.16). However, cue did not interact with attention [*F*(1, 63) = 0.38, *p* = 0.540, *η*_*p*_^2^ = 0.01].

To determine whether there were differences in participants’ ability to distinguish between studied and novel items, A′ was calculated for each participant using hit rates (i.e., correct identifications of presented items; *M* = 0.86, SD = 0.12) and false alarm rates (i.e., instances in which participants incorrectly identified a new item as having been presented; *M* = 0.25, SD = 0.22). Recognition performance as a function of cue and attention is shown in Fig. 4b. To examine differences in A′ on the recognition test, a 2 (cue: Friend, You) × 2 (attention: full, divided) mixed ANOVA did not reveal a main effect of cue [*F*(1, 63) = 1.50, *p* = 0.225, *η*_*p*_^2^ = 0.02] such that A′ was similar for You items (*M* = 0.88, SD = 0.13) and Friend items (*M* = 0.86, SD = 0.14). However, results revealed a main effect of attention [*F*(1, 63) = 7.48, *p* = 0.008, *η*_*p*_^2^ = 0.11] such that participants with full attention (*M* = 0.90, SD = 0.08) better recognized items than participants under divided attention (*M* = 0.83, SD = 0.13). Moreover, cue did not interact with attention [*F*(1, 63) = 0.41, *p* = 0.522, *η*_*p*_^2^ = 0.01].

Finally, to examine differences in confidence as a function of cue, a 2 (cue: Friend, You) × 2 (attention: full, divided) mixed ANOVA revealed a main effect of cue [*F*(1, 63) = 4.51, *p* = 0.038, *η*_*p*_^2^ = 0.07] such that participants were more confident in their recognition of You items (*M* = 85.27, SD = 17.01) than Friend items (*M* = 81.76, SD = 17.71). Additionally, results revealed a main effect of attention [*F*(1, 63) = 8.26, *p* = 0.006, *η*_*p*_^2^ = 0.12] such that participants with full attention (*M* = 79.12, SD = 14.44) were more confident than participants under divided attention (*M* = 68.18, SD = 17.97). However, cue did not interact with attention [*F*(1, 63) < 0.01, *p* = 0.962, *η*_*p*_^2^ < 0.01].

#### Discussion

In Experiment 3, despite overall recall deficits in participants under divided attention, these participants overcame this recall deficit and strategically allocated their remaining cognitive resources to remember goal-relevant information. Specifically, participants demonstrated intact goal-directed cognitive control, even when under divided attention, by showing better recall of items they were responsible for remembering compared to items their friend was responsible for remembering (but this effect was not observed on the subsequent recognition test, perhaps due to a ceiling effect). Thus, although our instinct is often to attempt to remember as much information as possible, participants may have maximized memory utility by selectively rehearsing and remembering goal-relevant information, regardless of attention at encoding, consistent with engaging in responsible remembering.

## General discussion

Although people are generally aware of the costs of divided attention (Calderwood et al., 2014), we are frequently exposed to situations where our attentional resources are divided and when attention is divided, the ability to engage in responsible remembering may be impaired. However, in certain situations, people may be able to employ strategic cognitive operations to counteract the costs of reduced attentional resources. These instances exemplify what we are calling *responsible attention*: the allotment of attentional resources that are needed to engage in responsible remembering as well as the cognitive mechanisms that are preserved or even enhanced under divided attention.

In the present study, we presented participants with situations involving responsible remembering and compared participants completing the tasks under full and divided attention. In Experiment 1, we presented participants with words paired with point values counting towards their score if recalled. However, participants only received the points for recalling a word if they bet on it in the study phase; if they bet on a word but failed to recall it, they lost the associated points (adapted from McGillivray & Castel, 2011). Thus, learners had to balance seeking gains and minimizing losses (see Murphy & Knowlton, 2022 for an illustration of the effect of framing goals in terms of maximizing gains or minimizing losses). Results revealed that participants under divided attention were less able to engage in responsible remembering by strategically betting on and remembering the most valuable information. Therefore, although some forms of selective memory can be preserved under divided attention (see Middlebrooks et al., 2017), introducing consequences for forgetting impaired selectivity in participants under divided attention, indicating the crucial role of attention in participants’ ability to remember important information.

It is interesting and important to note that healthy older adults in prior work (Fourquet et al., 2020; McGillivray & Castel, 2011; Siegel & Castel, 2019) performed well on a similar “betting” task (especially after task experience), whereas in the present work, when attention was divided for younger adults (which often can lead to comparable performance to that of older adults under full attention, see Castel & Craik, 2003), this led to some impairments. Older adults may become more attuned regarding the need to be selective and responsible regarding betting, especially after some task experience, as older adults may be aware of the consequences of forgetting and the challenge of remembering specific information (see also Murphy & Castel, 2022a). In contrast, younger adults may be more prone to focusing on larger amounts of information (and betting on more items), and when under divided attention, may not sufficiently consider the potential costs this has on selective learning (e.g., Barnes & Dougherty, 2007; see also Peng & Tullis, 2021). Thus, the differences between older adults and younger adults under divided attention may be driven by older adults’ experience with memory challenges (and adapting betting behavior accordingly) whereas younger adults may not initially appreciate how divided attention can impact memory and metacognitive accuracy in a selective memory task. However, future research is needed to further clarify these potential age-related differences as they may reflect changes individual differences in fluid intelligence (e.g., Murphy et al., 2021) or more strategic uses of memory (see Knowlton & Castel, 2022) by younger and older adults.

In Experiment 2, we presented participants with unassociated word pairs and solicited metacognitive predictions of recall (i.e., JOLs). Some theories of metacognition suggest that the cognitive resources necessary for metacognitive monitoring may result in impaired accuracy when attention is divided (e.g., Nelson & Narens, 1990) while others suggest that metacognition may demand few attentional resources, preserving the accuracy of metacognitive monitoring under divided attention (e.g., Boekaerts & Niemivirta, 2000; Peng & Tullis, 2021). Contrary to previous work suggesting that some aspects of metacognition are hindered under divided attention (e.g., Beaman et al., 2014; Kelley & Sahakyan, 2003; Konishi et al., 2021; Sacher et al., 2009, 2013), results revealed that the relative accuracy of metacognitive judgments was preserved or even enhanced when studying under divided attention (consistent with Peng & Tullis, 2021).

Although we expected divided attention to result in less relatively accurate JOLs, when participants’ attention is divided, they may become more metacognitively aware of what will be later remembered. Monitoring accuracy is driven by the diagnosticity of the cues that learners attend to when making judgments and according to the cue utilization framework (Koriat, 1997), differences in judgment accuracy for participants under divided attention may arise from differences in cue utilization. Specifically, reduced attentional resources during encoding may draw learners’ attention to different cues (that are potentially more diagnostic of memory) than they would otherwise normally attend to during monitoring. Thus, enhanced metacognitive accuracy in Experiment 2 may have arisen as an unexpected side effect of divided attention if the strength of each memory trace became more apparent when studying with fewer attentional resources available. However, future work should solicit recollection responses (i.e., “remember” judgments or vividness ratings; see Gardiner et al., 1998; Johnson et al., 1993) to better understand what cues learners are basing their metacognitive judgments on when their attention is divided and how participants under divided attention can sometimes be more relatively accurate in their metacognitive monitoring.

The conflicting effects of divided attention on metacognitive monitoring in Experiment 1 (impaired) and Experiment 2 (preserved/enhanced) may be attributable to the consequences of forgetting as well as the binary judgments used in Experiment 1. Specifically, the binary form of metacognitive monitoring (i.e., betting) when there were consequences for forgetting may have caused learners to exercise more conservative monitoring of their learning whereas participants estimating the probability of remembering word pairs may have focused more on the cues that influence memorability. Thus, there may be some instances where fewer attentional resources can result in impaired or enhanced metacognition and responsible remembering (i.e., remembering what one says they will remember).

In Experiment 3, rather than examining selectivity and metacognition, we investigated whether participants could still implement strategic cognitive control mechanisms to remember goal-relevant information at the expense of information that could potentially be offloaded (i.e., *responsible forgetting*). Specifically, participants were presented with a list of items to pack for a camping trip with half of the items designated as items the participant should remember and half of the items designated as an item their hypothetical friend should remember. Similar to previous work (Murphy & Castel, 2021b), results revealed that participants were similarly selective for goal-relevant information regardless of the amount of attention and resources available during encoding. Thus, both participants under full and divided attention maximized memory utility by strategically remembering goal-relevant information, indicating that some forms of cognitive control can be preserved under divided attention.

Compared with Experiment 1 in which we measured participants’ ability to selectively remember valuable information, Experiment 3 provides a measure of responsible forgetting: the strategic forgetting of information to maximize memory utility (see Murphy & Castel, 2021b). Since participants’ ability to engage in strategic encoding was impaired under divided attention but strategic forgetting was preserved when fewer attentional resources were available, these different processes contributing to responsible remembering may be differentially impacted by divided attention during encoding. However, future work should examine the potential boundary conditions of responsible remembering and how learners strategically remember and forget information depending on whether to-be-remembered information is paired with point values or using more real-world scenarios.

Collectively, the present study indicates that there are some situations where responsible remembering can be impaired without a full allotment of attentional resources (i.e., situations with rewards and consequences for remembering and forgetting). Additionally, there are instances where responsible remembering can be enhanced when fewer attentional resources are available (i.e., when predicting what will be later remembered). Finally, certain metacognitive mechanisms are preserved when under divided attention (i.e., strategically prioritizing goal-relevant information at the expense of information that could be offloaded). Thus, we provide evidence for *responsible attention* such that the allowance of attentional resources during encoding can impact one’s ability to engage in responsible remembering. Specifically, responsible attention aims to account for why and when participants sometimes show memory and metamemory deficits under divided attention but other times show maintenance or benefits.

The notion of responsible attention, a facet of the responsible remembering framework, encompasses attention-based boundary conditions to engaging in responsible remembering by illustrating the allotment of attentional resources required to successfully engage in responsible remembering in different contexts. In some situations, full attention is required (i.e., executing strategic metacognitive and encoding mechanisms to remember valuable information) whereas other cognitive functions contributing to responsible remembering (i.e., metacognitive monitoring or strategic forgetting) are less dependent on the availability of cognitive resources. However, future work should investigate responsible attention in situations when the availability of attentional resources is not under experimenter control as well as how participants utilize their attentional resources. Specifically, future research could examine if participants are aware of and responsive to the attentional resources necessary to engage in responsible remembering.

Although we were primarily interested in the effect of divided attention on metacognitive accuracy and learning outcomes, responsible remembering may be affected by the degree to which the learner is engaged with the distractor. Specifically, divided attention may lead to responsible remembering if people become aware that attention is limited and subsequently selectively focus on important items. However, a strategy for accomplishing this could be to strategically allocate attention towards remembering important information and neglect whatever is dividing your attention. For example, if participants become aware that the division of their attention is harming their memory for important information or information with consequences if forgotten, they could shift their attention towards these items (and perhaps ignore the secondary task) to maximize memory utility. However, in the present study, we excluded participants who did not engage with the secondary task, and divided attention task performance did not relate to other performance measures (see Tables 1, 2, and 3) but future work should examine the strategies participants use to achieve desirable memory outcomes under divided attention.

In sum, we evaluated the role of attentional resources (as measured via divided attention during encoding) on measures of responsible remembering and demonstrated that responsible remembering can be impaired, enhanced, or preserved under divided attention in certain contexts. Although a full allotment of attention may be necessary to remember items of importance or with consequences if forgotten, metacognitive accuracy may be enhanced under certain divided attention conditions. Additionally, responsible rememberers can efficiently utilize cognitive control mechanisms to remember goal-relevant information, even when under divided attention during encoding. Thus, people should learn to mitigate the effects of divided attention (perhaps via a metacognitive awareness of their reduced attentional and memory resources) to more efficiently allocate their remaining attentional resources towards valuable information by more selectively focusing on the most important information at the expense of less important information.

## Footnotes

1 In cases where recall and/or judgments were constant, Gamma correlations could not be computed. This resulted in 29 participants with empty cells in this mixed ANOVA.
